# Real-world management of tuberous sclerosis complex–associated renal angiomyolipomas: the impact of mTOR inhibitors

**DOI:** 10.1093/ckj/sfag072

**Published:** 2026-03-03

**Authors:** Nicolas Gemander, Myriam Dao, Emilie Kalbacher, Jean-Christophe Fantoni, François Provot, Jean-Michel Correas, Dominique Joly

**Affiliations:** Department of Nephrology, Dialysis and Renal Transplantation, Hôpital Universitaire de Bruxelles (H.U.B.), Erasme Hospital, Université libre de Bruxelles (ULB), Brussels, Belgium; Department of Nephrology, Hôpital Necker–Enfants Malades, Assistance Publique–Hôpitaux de Paris (APHP), Université Paris Cité, Paris, France; Department of Nephrology, Hôpital Edouard Herriot, Hospices Civils de Lyon, Lyon, France; Department of Urology, Hôpital Claude Huriez, Centre Hospitalier Universitaire (CHU) de Lille, Lille, France; Department of Nephrology, Hôpital Claude Huriez, CHU de Lille, Lille, France; Department of Radiology, Hôpital Necker–Enfants Malades, APHP, Université Paris Cité, Paris, France; Department of Nephrology, Hôpital Necker–Enfants Malades, Assistance Publique–Hôpitaux de Paris (APHP), Université Paris Cité, Paris, France; F-CRIN INI-CRCT (Cardiovascular and Renal Clinical Trialists), Nancy, France

**Keywords:** mTOR inhibitors, renal angiomyolipoma, renal haemorrhage, selective arterial embolization, tuberous sclerosis complex

## Abstract

**Background:**

Renal angiomyolipomas (AMLs) drive substantial morbidity in tuberous sclerosis complex (TSC) through haemorrhage and repeated invasive procedures. While mammalian target of rapamycin inhibitors (mTORis) reduce AML volume, long-term real-world data on clinically meaningful bleeding and procedure outcomes remain limited, particularly in cohorts enriched for high-risk imaging phenotypes.

**Methods:**

We conducted a multicentre observational study (2004–2020) in three French tertiary centres. Among 103 included patients, the 96 with TSC constituted the primary analysis set. We assessed AML-related haemorrhage and selective arterial embolization (SAE). For patients initiating mTORi, follow-up was split into pre- and post-initiation periods and incidence rate ratios (IRRs) were estimated using Poisson regression with patient-time offsets and patient-clustered robust standard errors; generalized estimating equation Poisson and negative binomial models were used as sensitivity analyses.

**Results:**

Total follow-up was 694.3 patient-years. Thirty-nine patients (40.6%) initiated an mTORi (everolimus 94.9%). Haemorrhage rates decreased from 0.061 to 0.007 events per patient-year after mTORi initiation {IRR 0.11 [95% confidence interval (CI) 0.02–0.62]} and SAE rates decreased from 0.197 to 0.020 sessions per patient-year [IRR 0.10 (95% CI 0.04–0.29)]. Therapeutic inertia remained substantial: among 57 never-treated patients, high-risk imaging features and active complications were frequent at the last assessment.

**Conclusions:**

In this long-term real-world cohort of TSC-associated renal AMLs, mTORi initiation was associated with markedly lower rates of AML haemorrhage and SAE. Despite these benefits, many high-risk patients remained untreated in routine care, supporting earlier nephrology referral, systematic risk stratification and timely treatment initiation to reduce preventable AML-related morbidity.

## Graphical Abstract

**Graphical Abstract:**
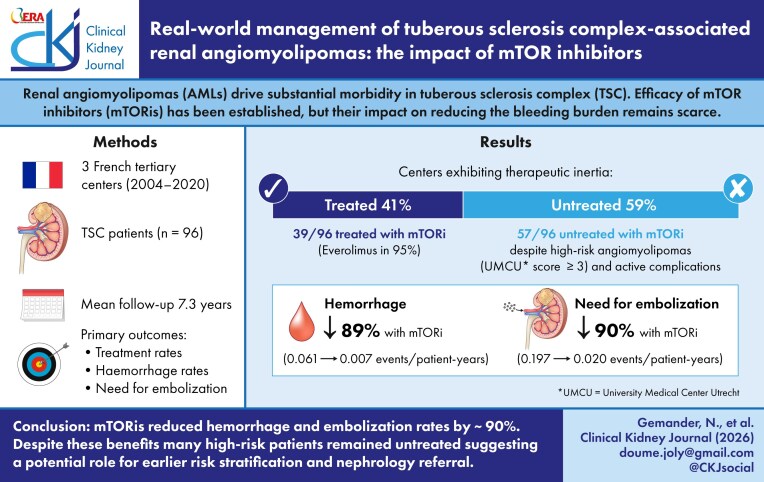

KEY LEARNING POINTS **What was known:**

- Renal angiomyolipomas (AMLs) are the leading cause of morbidity in tuberous sclerosis complex (TSC), primarily due to haemorrhagic risks and the need for invasive procedures.
- While mammalian target of rapamycin inhibitors (mTORis) are known to reduce tumour volume, real-world evidence regarding their ability to prevent hard outcomes—specifically haemorrhage and embolization—in high-risk populations remains limited.

**This study adds:**

- In the multicentre OSCAR cohort, initiation of mTORis was associated with a 10-fold reduction (≈90%) in both haemorrhagic events and the need for selective arterial embolization.
- Therapeutic inertia is substantial: nearly 60% of patients remained untreated despite presenting with high-risk AMLs [University Medical Center Utrecht (UMCU) stage ≥3] and active complications.
- We demonstrate that medical therapy effectively mitigates the ‘bleeding burden’ even in a population with a history of severe renal involvement.

**Potential impact:**

- These findings highlight the urgency of overcoming therapeutic inertia through earlier nephrology referral and systematic risk stratification using the UMCU classification.
- Timely initiation of mTORis in high-risk patients should be prioritized to prevent life-threatening haemorrhage and avoid nephron-damaging invasive interventions.

## INTRODUCTION

Tuberous sclerosis complex (TSC) is a rare autosomal dominant disorder caused by loss-of-function germline mutations in either of the tumour-suppressor genes *TSC1* (encoding hamartin) or *TSC2* (encoding tuberin) [1]. The majority of cases result from *de novo* mutations [2]. Hamartin and tuberin form a complex to inhibit the mammalian target of rapamycin (mTOR) pathway, thereby regulating protein synthesis, cell growth, proliferation and angiogenesis [2]. A loss of function in either the *TSC1* or *TSC2* gene leads to activation of the mTOR pathway, which promotes the development of hamartomas that can affect virtually any organ [3].

In the kidneys, TSC is frequently associated with multiple, bilateral angiomyolipomas (AMLs). These benign tumours, composed of blood vessels, smooth muscle cells, epithelioid cells and fat, typically develop during childhood and adolescence and progress over time [4, 5]. AMLs are a major cause of morbidity and represent the leading cause of mortality among adults with TSC [6]. Complications include rupture, which can cause life-threatening retroperitoneal haemorrhage [7–9], progressive compression of the urinary tract and replacement of normal parenchyma, resulting in kidney function loss [6, 10].

Most studies recommend preventive intervention for AMLs at high risk of bleeding, defined by a size >3.5 cm [11] or the presence of intralesional aneurysms >5 mm. Available treatments include surgery, arterial embolization, percutaneous ablation and mTOR inhibitors (mTORis) [12]. The first three approaches are potentially curative but invasive, whereas mTORis have a suspensive effect, reducing AML size while treatment is ongoing. For several years, renal AMLs ≥3 cm have been eligible for mTORi therapy [13]. Everolimus has been granted marketing authorization for this indication following a prospective randomized clinical trial versus placebo: its use reduces the volume of renal AMLs, and follow-up studies have shown a protective effect on renal function decline and bleeding risk [14, 15].

In this retrospective/prospective study, we report on a large series of adult patients referred to three tertiary centres for the management of multiple renal AMLs, regardless of TSC association. We describe under real-life conditions the management of AMLs and long-term renal outcomes, with a focus on the implementation of mTORis and their potential impact on the natural history of kidney disease.

## MATERIALS AND METHODS

### Cohort formation

We performed a multicentre retrospective/prospective observational study in three French tertiary centres (Paris, Lille and Lyon). Eligible participants were adults with either multiple renal AMLs (five or more lesions) or renal AMLs associated with TSC and at least one visit to the participating centre between January 2004 and August 2016. All patients were enrolled between August 2015 and August 2016. At inclusion, retrospective data were collected and prospective follow-up was established until August 2017 for Lille (28 patients) and Lyon (5 patients) and until August 2020 for Paris (70 patients). The primary analysis set consisted of patients with confirmed TSC; patients with sporadic multiple AMLs were described descriptively due to small numbers.

### Data sources and variables collected

Data were extracted from both paper-based and electronic medical records. We recorded date of birth, date of initial visit, date of inclusion, date of last contact and dates of each clinic visit, hospitalization, renal complications and renal interventions (see below).

Individual TSC characteristics recorded at inclusion were gender, genetic data, parental status, autonomy and extrarenal abnormalities associated with the diagnostic criteria of TSC [16]. All available kidney imaging results [ultrasound, computed tomography (CT) scan or magnetic resonance imaging (MRI)] since 2004 were recorded. Renal staging was centrally reviewed. Renal staging was determined using the University Medical Center Utrecht (UMCU) classification (Table 1) [17]. The presence and number of AMLs ≥3 cm were recorded.

**Table 1:** Renal angiomyolipoma staging for all patients, stratified by exposure to mTORis. Staging was based on the criteria proposed by the UMCU.^ [17]^ CT and MRI data were assessed at the time of mTORi initiation for treated patients or at the last available follow-up for untreated patients.

	Renal angiomyolipoma staging		TSC patients (*n* = 96)		Non-TSC patients (*n* = 7)
Stage	AMLs, *n*	AML size	At mTORi initiation (39 patients), *n* (%)	At last news (no mTORi initiation; 57 patients), *n* (%)	At last news, *n*
0	Any	All <1 cm	0	1 (2)	0
1/2	≤5 or >5	<3.5 cm	3(8)	16 (28)	2
3	≤5	At least one ≥3.5 cm	15 (38)	22 (39)	1
4/5	>5	<5 or ≥5 ≥3.5 cm	2 (5)	3 (5)	4
6	Kidney not recognizable (i.e. complete replacement o parenchyma by AMLs)		10 (26)	3 (5)	–
Undetermined/missing			3 (8)	9 (16)	1 (14)
Aneurysms (>5 mm)			8 (20)	5 (9)	–

All available serum creatinine values and corresponding estimated glomerular filtration rates (eGFRs) were collected, calculated using the Modification of Diet in Renal Disease or Chronic Kidney Disease Epidemiology Collaboration equations. Data on proteinuria, creatininuria and blood pressure were also recorded.

### Exposure to mTORis

Exposure to mTORis was defined as the initiation of any mTORi during follow-up. For incidence rate analyses, individual follow-up time was split into pre-initiation and post-initiation periods. A 90-day exposure lag was explored in sensitivity analyses to reduce protopathic bias (events occurring around treatment initiation). Adverse events potentially related to mTORis, treatment interruptions and therapeutic drug monitoring (trough levels) were extracted from medical records when available. Trough levels were summarized as patient-level means across recorded measurements. Adverse events were categorized from free-text clinical documentation (non-mutually exclusive categories) and reported descriptively.

### Outcomes

Renal complications were haemorrhagic complication of one or more AMLs, defined as acute symptomatic bleeding with radiologic or clinical confirmation, classified as requiring emergency intervention [selective arterial embolization (SAE)/surgery] or conservative management (self-limited or late diagnosis); urinary tract compression; deterioration of renal function (doubling of serum creatinine compared with the initial visit or the need for kidney dialysis or transplantation); renal infection (temperature >38.5°C associated with urinary tract infection); diagnosis of malignant renal lesions and death (all causes). Renal interventions were SAE (counted per session irrespective of kidney side), renal biopsy, surgery (partial or complete nephrectomy), minimally invasive interventions (cryotherapy, radiofrequency), antibiotic treatment, urinary drainage, initiation of periodic haemodialysis and kidney transplantation. For each procedure, both date and indication were documented.

We assessed AML-related haemorrhage and renal interventions, with a focus on SAE as the most frequent invasive renal procedure in this cohort. A composite ‘any renal event’ outcome included renal haemorrhage, urinary tract obstruction, renal infection, renal malignancy, renal function deterioration and death. For time-to-event analyses, the composite renal event was defined as the first occurrence of any renal complication or invasive renal procedure.

### Statistical methods

Descriptive statistics were used to summarize baseline characteristics. Continuous variables are reported as mean [standard deviation (SD)] or median (range) and categorical variables as *n* (%). Analyses were primarily performed in TSC patients.

Primary analyses focused on event and procedure rates to capture the real-world burden of recurrent AML-related outcomes. Annualized rates were calculated as the number of events divided by the total patient-years at risk and are presented with exact 95% Poisson confidence intervals (CIs); for groups with zero events, the upper 95% confidence bound was estimated using the rule of three. For patients initiating an mTORi, follow-up was split into pretreatment (from first visit to initiation) and post-treatment (from initiation to the last follow-up) periods.

To compare post- versus pretreatment rates while accounting for unequal follow-up durations and within-patient correlation of recurrent events, we fitted Poisson regression models with a log link and log(person-time) offset, using patient-level cluster-robust (sandwich) standard errors, and report incidence rate ratios (IRRs) with 95% CIs. Overdispersion was assessed and sensitivity analyses included generalized estimating equation (GEE) Poisson models (exchangeable working correlation) and negative binomial models with robust standard errors.

Secondary analyses evaluated time to first renal event using Kaplan–Meier methods and Cox proportional hazards models with time-dependent mTORi exposure (counting process start–stop formulation). A 90-day lag sensitivity analysis was performed to minimize potential surveillance bias around treatment initiation. All statistical analyses were performed using R version 4.2.0 (R Foundation for Statistical Computing, Vienna, Austria).

### Ethics approval and consent to participate

This study was approved by the French Ministry of Research (authorization no. 15.211). Prior to granting approval, the Ministry sought the opinion of an independent ethics committee, the Comité consultatif sur le traitement de l’information en matière de recherche (CCTIRS), which issued a favourable opinion. In accordance with French regulations for non-interventional studies, written informed consent is not required; however, patients must be given the opportunity to object to participation. For this study, non-opposition was obtained from all participants regarding the use of their de-identified medical records data.

## RESULTS

### Study cohort characteristics

A total of 103 patients were included; 96 had confirmed TSC and comprised the primary analysis set. The remaining seven patients had sporadic multiple AMLs and were analysed descriptively. Demographics and baseline characteristics are summarized in Table 2.

**Table 2:** Demographic, genetic, extrarenal and renal characteristics at inclusion for the overall cohort and for TSC and non-TSC patients separately.

Characteristics	Total (*N* = 103)	TSC (*n* = 96)	Non-TSC (*n* = 7)
Female, *n* (%)	69 (67.0)	64 (66.7)	5 (71.4)
Age at inclusion (years), mean ± SD	38.6 ± 12.6	37.7 ± 12.0	50.5 ± 15.3
Age at initial visit (years), mean ± SD	34.0 ± 12.8	33.2 ± 12.3	45.4 ± 15.0
Known mutation testing performed, *n* (%)	38 (36.9)	36 (37.5)	2 (28.6)
*TSC1* mutation, *n*	4	4	0
*TSC2* mutation, *n*	21	21	0
*PKD1-TSC2* deletion, *n*	2	2	0
No mutation detected, *n*	7	6	1
Skin lesions, *n* (%)	81 (78.6)	79 (82.3)	2 (28.6)
Neurological manifestations, *n* (%)	76 (73.8)	76 (79.2)	0 (0.0)
Epilepsy, *n* (%)	65 (63.1)	65 (67.7)	0 (0.0)
Subependymal giant cell astrocytoma, *n* (%)	34 (33.0)	34 (35.4)	0 (0.0)
TSC-associated neuropsychiatric disorders, *n* (%)	28 (27.2)	28 (29.2)	0 (0.0)
Severe intellectual disability requiring constant assistance, *n* (%)	22 (21.4)	22 (22.9)	0 (0.0)
Lymphangioleiomyomatosis, *n* (%)	31 (30.1)	30 (31.2)	1 (14.3)
Pneumothorax/chylothorax, *n* (%)	10 (9.7)	10 (10.4)	0 (0.0)
Ventilatory insufficiency, *n* (%)	4 (3.9)	4 (4.2)	0 (0.0)
Neuro-endocrine tumour, *n* (%)	5 (4.9)	5 (5.2)	0 (0.0)
Retinal lesion, *n* (%)	12 (11.7)	12 (12.5)	0 (0.0)
Cardiac rhabdomyoma, *n* (%)	13 (12.6)	13 (13.5)	0 (0.0)
Hypertension at inclusion visit, *n* (%)	21 (20.4)	20 (20.8)	1 (14.3)
eGFR <60 ml/min/1.73 m^2^ at inclusion visit, *n* (%)	3/26 (11.5)	3/24 (12.5)	0/2 (0.0)
Proteinuria available at inclusion visit, *n* (%)	13 (12.6)	12 (12.5)	1 (14.3)
Clinical proteinuria >0.5 g/g (inclusion visit), *n* (%)	1/13 (7.7)	1/12 (8.3)	0/1 (0.0)
Number of ≥3 cm AMLs per kidney (kidneys with ≥1 lesion)	Mean 2.1 ± 1.4; median 2 (range 1–7)	Mean 2.2 ± 1.4; median 2 (range 1–7)	Mean 1.8 ± 0.8; median 2 (range 1–3)

### Follow-up

In TSC patients, total follow-up was 694.3 patient-years (mean 7.23 ± 4.48 years). Thirty-nine patients initiated an mTORi during follow-up, contributing 198.0 patient-years pre-initiation and 149.9 patient-years post-initiation; never-treated patients contributed 346.4 patient-years.

### Renal complications

Bleeding from an AML was the most common renal complication, affecting 11 patients (11.5%) with an average of 2.0 ± 1.2 episodes per patient (0.03 events per patient-year; Table 3). Overall, 22 haemorrhage episodes were recorded: 11 required emergency haemostasis (SAE, *n* = 9; surgery, *n* = 2) and 11 were conservatively managed (self-limited or diagnosed too late for emergency measures). Urinary obstruction occurred in five patients (5.2%, 0.013per patient-year), renal infection in five and renal function deterioration (doubling of serum creatinine) in six. Three patients (one *PKD1*/*TSC2* deletion, two *TSC2* mutations) progressed to end-stage renal disease and required haemodialysis; two later underwent kidney transplantation. Three patients died (astrocytoma, metastatic epithelioid AML, sudden death).

**Table 3:** Recurrent complications and invasive procedures during follow-up: crude rates and IRRs. Rates are expressed per patient-year with exact 95% Poisson CIs. IRR post versus pre was estimated using Poisson regression with log (person time) offset and patient-level cluster robust standard errors; sensitivity analyses with GEE Poisson and negative binomial models are shown in Supplementary Table S1.

Outcome/procedure	TSC total (*N* = 96)	TSC mTOR pre (*n* = 39)	TSC mTOR post (*n* = 39)	TSC no mTOR (*n* = 57)	Non-TSC total (*N* = 7)
Patient-years	694.3	198.0	149.9	346.4	56.8
Invasive procedures					
SAE					
Patients with procedure, *n* (%)	33 (34.4)	18 (46.2)	3 (7.7)	15 (26.3)	0 (0.0)
Procedures per patient, mean (SD)	2.0 (1.3)	2.2 (1.7)	1.0 (0.0)	1.6 (0.8)	0.0 (0.0)
Procedure rate, per patient-year	0.095	0.197	0.020	0.069	0.000
Microinvasive treatment					
Patients with procedure, *n* (%)	2 (2.1)	0 (0.0)	0 (0.0)	2 (3.5)	0 (0.0)
Procedures per patient, mean (SD)	1.5 (0.7)	0.0 (0.0)	0.0 (0.0)	1.5 (0.7)	0.0 (0.0)
Procedure rate, per patient-year	0.004	0.000	0.000	0.009	0.000
Renal surgery					
Patients with procedure, *n* (%)	6 (6.2)	2 (5.1)	1 (2.6)	3 (5.3)	0 (0.0)
Procedures per patient, mean (SD)	1.0 (0.0)	1.0 (0.0)	1.0 (0.0)	1.0 (0.0)	0.0 (0.0)
Procedure rate, per patient-year	0.009	0.010	0.007	0.009	0.000
Complications and events					
Bleeding of AML					
Patients with event, *n* (%)	11 (11.5)	6 (15.4)	1 (2.6)	5 (8.8)	0 (0.0)
Events per patient, mean (SD)	2.0 (1.2)	2.0 (0.9)	1.0 (0.0)	1.8 (1.3)	0.0 (0.0)
Event rate, per patient-year	0.032	0.061	0.007	0.026	0.000
Urinary tract obstruction					
Patients with event, *n* (%)	5 (5.2)	2 (5.1)	1 (2.6)	2 (3.5)	0 (0.0)
Mean events per patient (SD)	1.8 (1.1)	1.0 (0.0)	1.0 (0.0)	3.0 (0.0)	0.0 (0.0)
Event rate, per patient-year	0.013	0.010	0.007	0.017	0.000
Renal infection					
Patients with event, *n* (%)	5 (5.2)	1 (2.6)	1 (2.6)	3 (5.3)	0 (0.0)
Event rate, per patient-year	0.009	0.005	0.007	0.012	0.000
Renal cancer					
Patients with event, *n* (%)	2 (2.1)	0 (0.0)	0 (0.0)	2 (3.5)	0 (0.0)
Event rate, per patient-year	0.004	0.000	0.000	0.009	0.000
Renal function deterioration					
Patients with event, *n* (%)	6 (6.2)	0 (0.0)	2 (5.1)	4 (7.0)	0 (0.0)
Event rate, per patient-year	0.023	0.000	0.013	0.040	0.000
Renal transplantation or dialysis					
Patients with event, *n* (%)	3 (3.1)	0 (0.0)	0 (0.0)	3 (5.3)	0 (0.0)
Event rate, per patient-year	0.013	0.000	0.000	0.026	0.000
Death					
Patients with event, *n* (%)	3 (3.1)	0 (0.0)	0 (0.0)	3 (5.3)	0 (0.0)
Event rate, per patient-year	0.004	0.000	0.000	0.009	0.000

In the non-TSC AML group (*n* = 7), no kidney complications occurred during follow-up (Table 3). Preventive embolization was performed in two patients and two underwent radiofrequency ablation or cryotherapy.

### Renal interventions

Invasive procedures were performed in 39 of 96 (40.6%) TSC patients. Thirty-three patients had SAEs (2 ± 1.3 procedures per patient, 0.095 per patient-year), mostly for bleeding prevention of large AMLs (*n* = 51 sessions), urinary obstruction (5 sessions) or emergency post-haemorrhage (9 sessions). Renal surgery was performed in six patients: two for bleeding AMLs (one total, one partial nephrectomy), three for large obstructive AMLs (one total, two partial nephrectomies) and one for renal cancer (partial nephrectomy). Microinvasive treatments (cryotherapy, radiofrequency ablation) were performed in two patients (one for haemorrhage prevention, one for obstruction) (Table 3).

### Use of mTORis and renal outcomes

**Figure 1:**
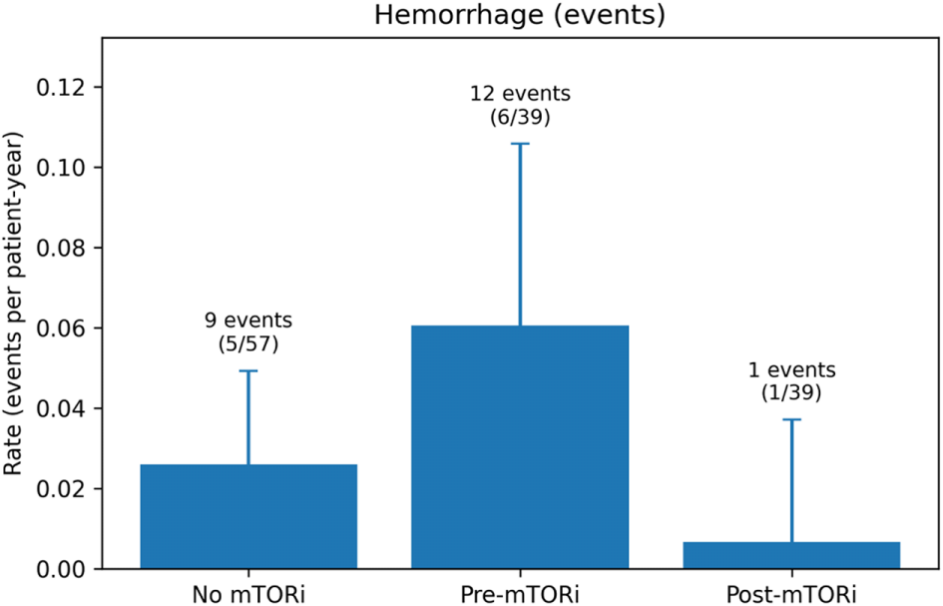
Annualized rates of AML haemorrhage and SAE according to mTORi exposure (TSC patients). Bars show crude rates of bleeding episodes with exact 95% Poisson CIs. For treated patients, follow-up was split into pretreatment and post-treatment periods. Numbers above bars indicate total events/sessions and the number of patients with one or more episodes (*n*/*N*).

**Figure 2:**
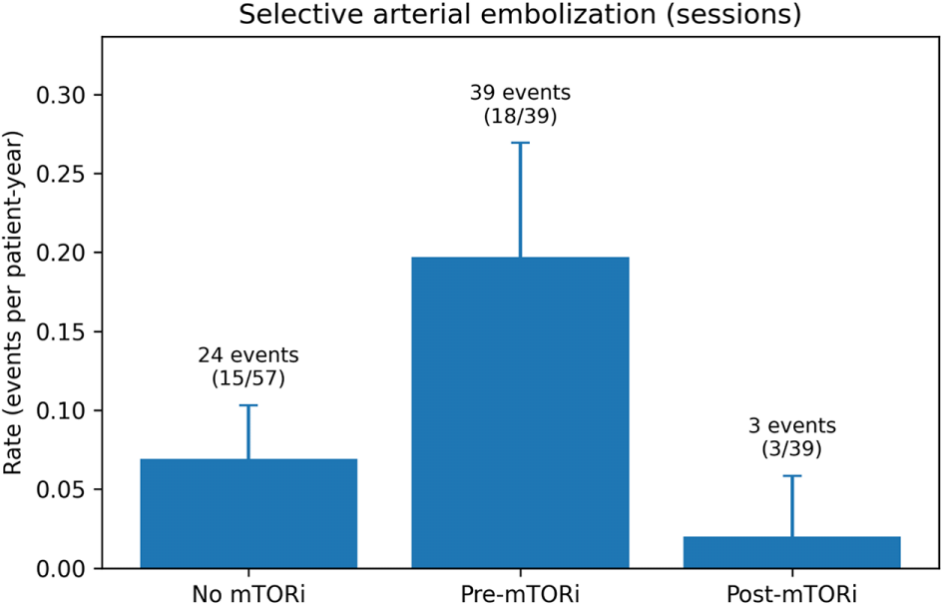
Annualized rates of SAE according to mTORi exposure (TSC patients). Bars show crude rates of sessions per patient-year with exact 95% Poisson CIs. For treated patients, follow-up was split into pretreatment and post-treatment periods. Numbers above bars indicate total events/sessions and the number of patients with one or more sessions (*n*/*N*).

Among TSC patients initiating an mTORi (*n* = 39), AML haemorrhage rates decreased from 0.061 events per patient-year pretreatment (12 events/198.0 per patient-years) to 0.0067 post-treatment (1 event/149.9 per patient-year) (Fig. 1). In Poisson regression models with log(person-time) offset and patient-level cluster-robust standard errors, post-treatment exposure was associated with a significantly lower haemorrhage rate [IRR 0.11 (95% CI 0.02–0.62); *P* = .012]. Similarly, SAE session rates decreased from 0.197 sessions/patient-year pretreatment (39 sessions/198.0 per patient-year) to 0.020 post-treatment (3 sessions/149.9 per patient-year), corresponding to an IRR of 0.10 (95% CI 0.04–0.29; *P* < .001)(Fig.2.). Results were consistent in GEE Poisson and negative binomial sensitivity analyses (Supplementary Table S1). Regarding tolerability, everolimus was the predominant agent (94.9%); adverse events were reported in 58.3% of patients (most frequently proteinuria and stomatitis), with treatment interruptions required in 21.7% of cases (Supplementary Table S3).

### Secondary time-to-event analyses

Kaplan–Meier estimates of remaining free of a first renal event (composite of renal complications or invasive renal procedures) were 87.4% at 1 year, 85.1% at 2 years, 78.5% at 5 years and 65.5% at 10 years (Supplementary Fig. S1). In time-dependent Cox models (Supplementary Table S2), the association between mTORi exposure and time to first renal event was not statistically significant, with wide CIs consistent with limited power. A 90-day exposure lag sensitivity analysis yielded directionally consistent estimates (hazard ratio <1) (Supplementary Table S2). Supplementary Fig. S2 (Simon–Makuch plot) provides a visual representation of event-free survival under time-varying mTORi exposure (with the 90-day lag), illustrating how patients contribute unexposed time before treatment and exposed time thereafter.

### Untreated high-risk patients

Among never-treated TSC patients (*n* = 57), haemorrhage and SAE rates remained clinically meaningful (0.026 and 0.069 per patient-year, respectively). A substantial proportion of never-treated patients fulfilled imaging-based high-risk criteria at the last follow-up, consistent with therapeutic inertia in routine care, despite the availability of effective medical therapy.

### Sporadic multiple AMLs (***n*** = 7)

In the sporadic multiple AML subgroup, no renal events were recorded during follow-up. Given the small sample size, no comparative analyses were undertaken.

## DISCUSSION

Although TSC was generally diagnosed in childhood and frequently accompanied by neurological disabilities, patients were typically referred for renal AML management much later, after age 30. At this initial visit, most already presented with bilateral, multiple AMLs ≥3 cm. This delay highlights the critical importance of early renal screening and referral, as recommended in current guidelines [18]. Two-thirds of TSC patients in this cohort were female. While recent large cohort studies have found no statistically significant association between sex and AML prevalence in TSC [17, 19], it remains possible that referral patterns are influenced by the perception of a higher bleeding risk in women [5].

Over a mean follow-up of 7.3 years, the clinical burden of TSC-associated renal AMLs was primarily driven by AML haemorrhage and the need for SAE, both of which occurred as recurrent outcomes. Haemorrhage—typically non-fatal—affected 11 of 96 TSC patients and was frequently recurrent (mean 2.0 ± 1.2 episodes among patients with bleeding), yielding an overall haemorrhage rate of 0.032 events per patient-year. In this setting, annualized rates provide a more faithful representation of morbidity than time-to-first-event curves, particularly in cohorts enriched for high-risk imaging phenotypes (UMCU stage ≥3) [7–9]. Accordingly, we compared pre- and post-mTORi periods using rate models with person-time offsets and patient-level robust standard errors, supported by GEE Poisson and negative binomial sensitivity analyses. We complemented these analyses with time-dependent Cox models including a 90-day exposure lag to reduce immortal time and surveillance biases around treatment initiation. Despite these approaches, residual confounding by indication and time-varying disease severity remain possible in an observational cohort.

The management of TSC-associated AMLs aims to prevent haemorrhage while preserving renal function and parenchyma. Across the study period, four preventive approaches were used: mTOR inhibition, SAE, nephron-sparing surgery and percutaneous ablation. The limited use of ablative techniques (cryoablation, radiofrequency, microwave) in this series mainly reflects evolving practice and availability during the study time frame; these modalities are now more widely implemented in our centres for selected cases—typically a limited number of AMLs measuring <5 cm—offering a potentially definitive alternative to surgery in appropriately selected patients [23, 24]. In contrast, SAE was commonly employed and remains a cornerstone for acute haemorrhage control and targeted prophylaxis in large AMLs. However, our experience confirms that repeat SAE is frequent, consistent with published series, [9, 20–22] likely reflecting multifocal disease and recurrence. Repeated embolizations may contribute to cumulative parenchymal loss and, over time, could be associated with progressive renal impairment.

In parallel, 39 TSC patients initiated an mTORi. Since 2012, these agents have been recommended as first-line therapy for asymptomatic, growing renal AMLs ≥3 cm [16]. This recommendation was supported by the phase 3 EXIST-2 trial (NCT00790400), demonstrating that mTORis reduce the volume of most renal AMLs [14, 15]. Importantly, the effect is largely suppressive, as tumours may regain volume after treatment discontinuation, underscoring the need for long-term therapy and structured monitoring in routine care.

Following mTORi initiation, we observed a marked reduction in haemorrhagic events and in the need for SAE, consistent with long-term follow-up data from EXIST-2 and registry evidence from the TuberOus SClerosis registry to increase disease Awareness (TOSCA) [15, 25]. Notably, these outcomes were observed in a population managed under real-life tertiary care conditions and appearing at relatively high baseline risk: most treated patients had high-risk imaging features at initiation (UMCU stage ≥3) and approximately one in six had a prior haemorrhage history. In comparison, EXIST-2 excluded patients with recent AML-related complications or interventions and prior haemorrhage was uncommon in the TOSCA AML substudy. Despite differences in study design and baseline risk, the concordant direction of effect across settings supports a clinically meaningful association between mTOR inhibition and reduced rates of AML-related complications and invasive procedures.

mTORis were well tolerated, with adverse events (mucositis, metabolic issues, proteinuria) consistent with the known safety profile. Most patients achieved target trough levels. Permanent discontinuation was notably lower in our series (2.9%) than in EXIST-2 (8.9%). This likely reflects strong patient motivation and our gradual dose escalation strategy (target 3–8 ng/ml). Long-term follow-up confirmed this favourable safety profile with no new signals [26]. Adverse events were collected as part of routine clinical care and were not systematically graded according to Common Terminology Criteria for Adverse Events (CTCAE) criteria. CTCAE-based grading was not mandated by the original study protocol and was inconsistently documented across centres and over time. In patients with severe cognitive impairment or limited ability to report symptoms, retrospective grading of toxicities such as mucositis or fatigue would have required subjective interpretation and was therefore considered unreliable. For this reason, adverse events were reported descriptively rather than retrospectively reclassified using CTCAE criteria. While perceived or anticipated treatment burden may have contributed to therapeutic inertia, the low rate of permanent discontinuation observed in our cohort suggests that real-world tolerability was generally acceptable. Most patients (57/96) did not receive mTORis. While one-third were low risk (UMCU stage 1–2), nearly half of the untreated group had high-risk AMLs (stage 3–6) yet experienced significant complications. Retrospectively, initiating therapy in this subgroup could likely have prevented these events and reduced the need for SAE. This undertreatment suggests ‘therapeutic inertia’ [27]. Importantly, therapeutic inertia was not a predefined objective of the study but emerged from the analysis of longitudinal management patterns; therefore, its determinants were not prospectively recorded. Although this was a non-interventional study and individual treatment rationales were not systematically documented, decisions were made within a limited number of expert multidisciplinary teams across the participating tertiary centres, allowing identification of recurring clinical considerations rather than isolated individual choices.

Several factors likely contributed. First, competing clinical priorities: in patients with severe neurological deficits, physicians may prioritize epilepsy management over asymptomatic renal lesions. Second, concerns regarding long-term treatment tolerance and monitoring burden—particularly in patients with pre-existing CKD or proteinuria—may have influenced the decision to defer therapy. Third, patient complexity: severe cognitive impairment (24%) complicates shared decision-making and adherence and caregivers may be reluctant to initiate chronic therapy for an often-asymptomatic condition. Finally, the transition from paediatric to adult care creates a vulnerability gap, often leading to delays in treatment initiation precisely when AMLs grow beyond the intervention threshold. Notably, treatment decisions were frequently reassessed over time and several initially untreated patients subsequently initiated mTORis once competing priorities stabilized or renal risk increased. Taken together, these elements suggest that therapeutic inertia reflects a multifactorial, context-dependent decision-making process rather than a uniform deviation from guideline recommendations.

This cohort also included seven patients with multiple renal AMLs without clinical or genetic criteria for TSC, analysed descriptively given the rarity and potential heterogeneity of sporadic multiple AMLs (including possible mosaic TSC). No haemorrhage or malignant transformation occurred during follow-up. These observations are hypothesis-generating only and management should remain individualized, as tumour size alone (e.g. a 4-cm threshold) should not automatically prompt intervention [28, 29].

In conclusion, our findings add to existing evidence that mTORis—beyond reducing AML volume—are associated with substantially lower rates of AML haemorrhage and the need for invasive procedures in TSC. Importantly, we observed persistent therapeutic inertia, with many patients who appeared to meet imaging-based eligibility criteria remaining untreated in real-life care. This reinforces the need for systematic renal risk assessment, early nephrological referral and proactive treatment initiation within a multidisciplinary care pathway.

## Supplementary Material

sfag072_Supplemental_File

## Data Availability

The data underlying this article cannot be shared publicly due to ethical and legal restrictions related to patient confidentiality. De-identified data may be made available from the corresponding author upon reasonable request, subject to institutional approval.
