# Supplementary material for: Real-world management of tuberous sclerosis complex–associated renal angiomyolipomas: the impact of mTOR inhibitors
Source: Clin Kidney J. 2026 Mar 3;19(4):sfag072. doi: 10.1093/ckj/sfag072 (PMC13076030; doi:10.1093/ckj/sfag072)

**Supplementary Table S1**. **Sensitivity analyses for incidence rate ratios (IRR) comparing post‑ versus pre‑mTOR inhibitor periods in treated TSC patients.**

**Incidence rate ratios (IRR) were estimated for AML hemorrhage episodes and selective arterial embolization (SAE) sessions in tuberous sclerosis complex (TSC) patients who initiated an mTOR inhibitor (n=39). Follow‑up time was split into pre‑treatment (from first visit to mTOR inhibitor initiation) and post‑treatment (from initiation to last follow‑up) periods. Event rates were modelled using (i) Poisson regression with a log link and log(person‑time) offset with patient‑clustered robust (sandwich) standard errors, (ii) GEE (generalized estimating equations) Poisson models with an exchangeable working correlation and robust standard errors, and (iii) negative binomial models with patient‑clustered robust standard errors.**

**IRRs represent the post‑ versus pre‑treatment rate ratio. Overdispersion diagnostics are provided as the Pearson chi‑square/df for Poisson models and the dispersion parameter (alpha) for negative binomial models. Two‑sided p‑values are reported.**

| Outcome | Model | IRR post vs pre | p | Overdispersion (Pearson/df) |
| --- | --- | --- | --- | --- |
| Hemorrhage | Poisson + cluster-robust SE | 0.11 (0.02–0.62) | 0.01217443 | 1.710104346 |
| Hemorrhage | GEE Poisson (exchangeable) | 0.12 (0.02–0.75) | 0.024068404 |  |
| Hemorrhage | Negative binomial + cluster-robust SE (alpha=0.00) | 0.11 (0.02–0.63) | 0.012801631 |  |
| SAE | Poisson + cluster-robust SE | 0.10 (0.04–0.29) | 2.29305E-05 | 1.773649297 |
| SAE | GEE Poisson (exchangeable) | 0.06 (0.01–0.26) | 0.000177336 |  |
| SAE | Negative binomial + cluster-robust SE (alpha=1.25) | 0.08 (0.03–0.21) | 5.13513E-07 |  |

**Supplementary Table S2**. **Time-dependent Cox proportional hazards models assessing the association between mTOR inhibitor exposure and time to first renal event, with a 90-day exposure lag sensitivity analysis.**

Models were fitted in patients with tuberous sclerosis complex (TSC; n=96) using a start–stop (counting-process) formulation and robust (sandwich) variance estimates to account for within-patient correlation induced by splitting follow-up into unexposed and exposed intervals. The composite renal event endpoint was defined as the first occurrence of any renal complication (AML hemorrhage, urinary tract obstruction, renal infection, renal malignancy, renal function deterioration, or death) or any invasive renal procedure (selective arterial embolization, renal surgery, or percutaneous ablation). mTOR inhibitor exposure was modelled as a time-dependent covariate (unlagged models: exposed from treatment initiation; lagged models: exposed from 90 days after initiation) to reduce potential immortal-time and surveillance bias around treatment initiation. *Adjusted models include age at follow-up start (per 10-year increase) and sex. †For the first-hemorrhage endpoint with a 90-day lag, no hemorrhagic events occurred during lagged exposed time; therefore, the hazard ratio could not be estimated.

| **Endpoint** | **Cox model** | | | **Adjustment** | **Covariate** | | **HR (95% CI)** | | | **p value** |  |
| --- | --- | --- | --- | --- | --- | --- | --- | --- | --- | --- | --- |
| A. Time to first renal event | Primary (time-dependent, no lag) | | | Unadjusted | mTOR inhibitor exposure (time-dependent) | | 0.82 (0.22–3.09) | | | 0.767 |  |
| A. Time to first renal event | Sensitivity (time-dependent, 90-day lag) | | | Unadjusted | mTOR inhibitor exposure (time-dependent) | | 0.25 (0.03–2.06) | | | 0.200 |  |
| A. Time to first renal event | Primary (time-dependent, no lag) | | | Adjusted* | mTOR inhibitor exposure (time-dependent) | | 0.78 (0.21–2.94) | | | 0.719 |  |
| A. Time to first renal event | Primary (time-dependent, no lag) | | | Adjusted* | Age at follow-up start (per 10 years) | | 1.20 (0.87–1.65) | | | 0.265 |  |
| A. Time to first renal event | Primary (time-dependent, no lag) | | | Adjusted* | Female sex | | 2.53 (0.93–6.86) | | | 0.068 |  |
| A. Time to first renal event | Sensitivity (time-dependent, 90-day lag) | | | Adjusted* | mTOR inhibitor exposure (time-dependent) | | 0.25 (0.03–2.04) | | | 0.194 |  |
| A. Time to first renal event | Sensitivity (time-dependent, 90-day lag) | | | Adjusted* | Age at follow-up start (per 10 years) | | 1.19 (0.86–1.64) | | | 0.298 |  |
| A. Time to first renal event | Sensitivity (time-dependent, 90-day lag) | | | Adjusted* | Female sex | | 2.60 (0.95–7.10) | | | 0.063 |  |
| B. Time to first AML hemorrhage | | Sensitivity (time-dependent, 90-day lag) | Unadjusted | | | mTOR inhibitor exposure (time-dependent) | | Not estimable† | — | | |
| B. Time to first AML hemorrhage | | Sensitivity (time-dependent, 90-day lag) | Adjusted* | | | mTOR inhibitor exposure (time-dependent) | | Not estimable† | — | | |

| Patients treated with an mTOR inhibitor (TSC) | n = 39 |
| --- | --- |
| mTOR inhibitor | everolimus: 37/39 (94.9%) sirolimus: 2/39 (5.1%) |
| Post-initiation follow-up (years) | 3.8 ± 2.8; |
| Therapeutic drug monitoring available (≥1 trough level) | 22/39 (56.4%) |
| Number of trough levels per patient (among those monitored) | median 2.5 (IQR 1–4) |
| Mean trough level (ng/mL) (patient-level mean) | 4.87 ± 2.52 |
| Mean trough level within target 3–8 ng/mL | 14/22 (63.6%) |
| Interruption information available | 23/39 (59.0%) |
| ≥1 treatment interruption reported | 5/23 (21.7%) |
| Duration of first interruption (months) | n=4; median 15 (2–24) |
| Second interruption reported | 1 patient |
| Adverse event information available | 24/39 (61.5%) |
| Any adverse event reported | 14/24 (58.3%) |
| • Proteinuria / nephrotic syndrome | 5/24 (20.8%) |
| • Stomatitis/mucositis | 4/24 (16.7%) |
| • Dyslipidemia | 4/24 (16.7%) |
| • Anemia | 2/24 (8.3%) |
| • Cytopenia (e.g., neutropenia) | 2/24 (8.3%) |
| • Psychiatric/neurobehavioral symptoms | 2/24 (8.3%) |
| • Hypophosphatemia | 1/24 (4.2%) |
| • Skin toxicity (rash/ulceration) | 1/24 (4.2%) |
| • Fatigue/asthenia | 1/24 (4.2%) |
| • Infertility | 1/24 (4.2%) |
| • Other | 1/24 (4.2%) |

**Table S3. mTOR inhibitor exposure, therapeutic drug monitoring, treatment interruptions and adverse events in TSC patients treated with an mTOR inhibitor.**

**Supplementary Figure S1.** **Kaplan-Meier estimate of the probability of remaining free of a first renal event.** The solid line represents the event-free survival probability over time (years since first visit). The shaded area indicates the 95% confidence interval (95% CI).


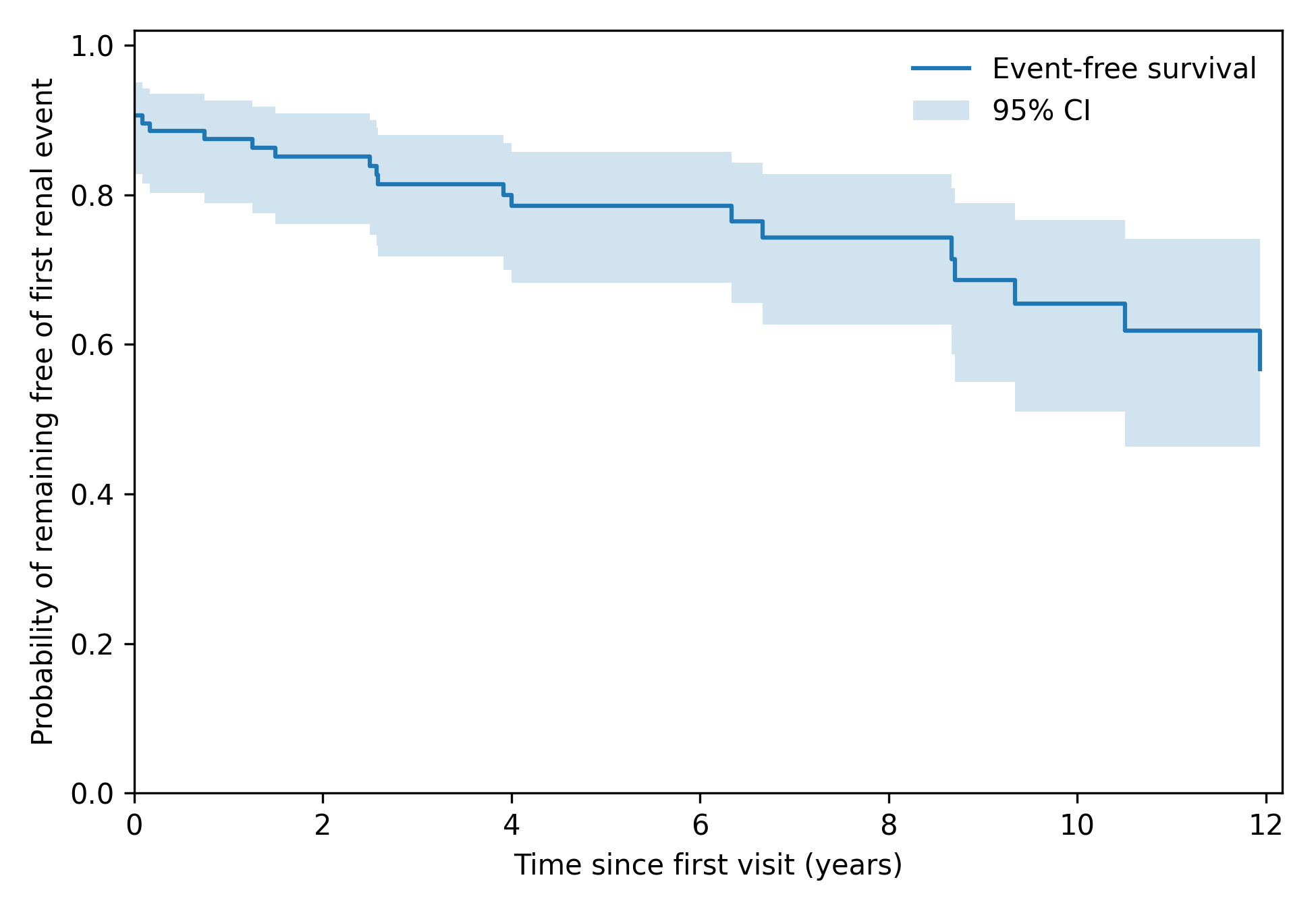


**Supplementary Figure S2.** **Simon–Makuch plot of renal event-free survival adjusted for time-dependent mTORi exposure (90-day lag sensitivity analysis).** In this analysis, mTORi exposure is treated as a time-dependent variable. Patients contribute person-time to the "unexposed" group until 90 days after treatment initiation, at which point they switch to the "exposed" group. This lag accounts for the potential delay in therapeutic effect.


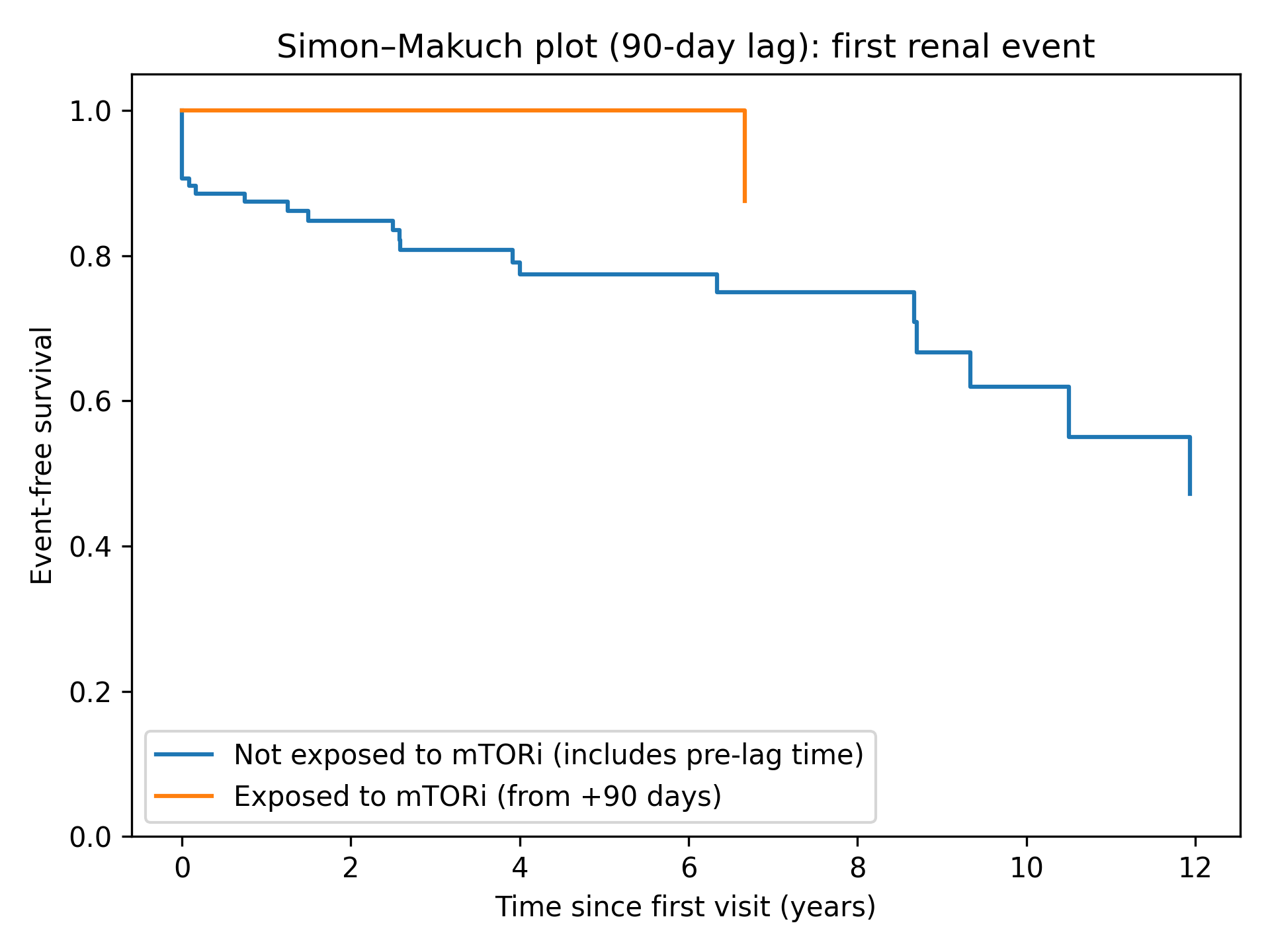

Supplement: sfag072_Supplemental_File [file sfag072_supplemental_file.docx]
